# Cytotoxicity Evaluation of Unmodified Paddlewheel Dirhodium(II,II)-Acetate/-Formamidinate Complexes and Their Axially Modified Low-Valent Metallodendrimers

**DOI:** 10.3390/molecules28062671

**Published:** 2023-03-15

**Authors:** Stephen de Doncker, Eva Fischer-Fodor, Cătălin Ioan Vlad, Patriciu Achimas-Cadariu, Gregory S. Smith, Siyabonga Ngubane

**Affiliations:** 1 Department of Chemistry, University of Cape Town, Rondebosch, Cape Town 7701, South Africa; 2 Tumour Biology Department, The Oncology Institute “Prof. Dr. Ion Chiricuţă”, 400015 Cluj-Napoca, Romania; 3 Department of Surgery, The Oncology Institute “Prof. Dr. Ion Chiricuţă”, 400015 Cluj-Napoca, Romania; 4 Department of Surgery and Gynecological Oncology, “Iuliu Hatieganu” University of Medicine and Pharmacy, 400337 Cluj-Napoca, Romania

**Keywords:** metallodrug, dirhodium(II,II), formamidine, cytotoxicity, metallodendrimers

## Abstract

Two diphenyl formamidine ligands, four dirhodium(II,II) complexes, and three axially modified low-valent dirhodium(II,II) metallodendrimers were synthesized and evaluated as anticancer agents against the A2780, A2780*cis*, and OVCAR-3 human ovarian cancer cell lines. The dirhodium(II,II) complexes show moderate cytotoxic activity in the tested tumor cell lines, with acetate and methyl-substituted formamidinate compounds displaying increased cytotoxicity that is relative to cisplatin in the A2780*cis* cisplatin resistant cell line. Additionally, methyl- and fluoro-substituted formamidinate complexes showed comparable and increased cytotoxic activity in the OVCAR-3 cell line when compared to cisplatin. The low-valent metallodendrimers show some activity, but a general decrease in cytotoxicity was observed when compared to the precursor complexes in all but one case, which is where the more active acetate-derived metallodendrimer showed a lower IC_50_ value in the OVCAR-3 cell line in comparison with the dirhodium(II,II) tetraacetate.

## 1. Introduction

The success of cisplatin and related platinum-based chemotherapies in the treatment of cancer has pioneered the development and study of various inorganic and organometallic compounds, such as cytotoxic agents [1]. Metal-containing compounds have favorable chemical properties that are not exhibited by purely organic compounds, including multiple oxidation states, various geometries, and the ability for conjugation to bioactive ligands, possibly leading to multiple modes of action, which may be exploited for use in medicinal chemistry [2]. The resistance displayed by malignancies toward platinum(II) metallodrugs, coupled with the inherent lack of specificity of platinum-containing compounds has become prevalent in recent times [3]. To circumvent the undesirable shortcomings of known platinum-based chemotherapies, the development of new metal-based drugs containing other platinum group metals has become increasingly prominent [4].

Consequently, potential drug leads containing metals, such as rhenium, ruthenium and rhodium, have been at the forefront for development as anti-tumor agents with some success [5,6,7,8,9,10,11,12]. The documented activity of mononuclear rhodium complexes showing cytotoxicity against both cisplatin-resistant and sensitive cell lines is evident in the literature [13]. Among the various strategies to improve selectivity, multinuclearity is often used to selectively target cancer cells through a phenomenon known as the enhanced permeability and retention (EPR) effect [14]. The enhanced metabolic requirements for the proliferation of cancer cells allows for the uptake of larger molecules when compared to healthy cells. This is exemplified by the trinuclear platinum-based compound BBR3464 showing an increased response toward cisplatin-resistant ovarian cancer [15]. Dendritic structures incorporating metal complex-based motifs are well reported, with general trends such as enhanced cytotoxicity and selectivity being observed for polynuclear compounds over their mononuclear analogs [16,17,18,19,20].

Dirhodium(II,II) complexes are of interest in this field of research and possess unique physicochemical properties, such as a metal–metal bonded binuclear core, the presence of vacant axial sites for further ligand occupancy, and the accessibility of a wider range of oxidation states when compared to mononuclear rhodium complexes [21]. In the decades following their inception, dirhodium(II,II) complexes have shown in vitro antiproliferation effects in cell lines, such as leukemia L1210, as well as sarcoma 180 and P388 [22,23,24,25,26,27]. Initially, it was demonstrated that dirhodium(II,II) acetate complexes bind covalently to DNA nucleotide bases via the axial binding site in a manner akin to cisplatin, alluding to a similar mechanism of action [28,29]. Additionally, their structural diversity, electronics, and axial site reactivity allow for binding to a variety of biologically relevant enzymes and molecules that are responsible for intracellular detoxification, such as glutathione [30]. Furthermore, the nature of the bridging ligands has been reported to affect the means of DNA interaction. For example, ligands bearing extended π-electron systems have been shown to bind to DNA via intercalation [31]. The incorporation of bridging ligands containing nitrogen donor atoms in homoleptic dirhodium (II,II) formamidinate complexes has also been explored utilizing Yoshida ascites sarcoma cells, which has been observed with no appreciable cytotoxic effects [32]. Furthermore, an in vivo study showed no appreciable general toxicity in rats at a dosage of 150 mg per test subject’s unit bodyweight in Kg [32]. To this end, heteroleptic dirhodium(II,II) complexes bearing formamidinate and acetate ligands have been shown to decrease toxicity toward healthy cells in comparison with homoleptic dirhodium(II,II) acetate complexes [33,34].

Herein, we report the synthesis of three new axially modified low-valent dirhodium (II,II) metallodendrimers containing dirhodium(II,II) complexes, bound via a single axial coordination site. The dendritic structure may allow for increased selective cellular uptake, alongside a multivalent drug delivery approach. The synthesized compounds were evaluated as potential antitumor agents against the A2780 human ovarian cancer cell line, the cisplatin resistant A2780*cis* human ovarian cancer cell line, and the highly prolific OVCAR-3 human ovarian cancer cell line. These were then compared to the toxicity profiles of ligands (**1**, **2**); the precursor complexes containing acetate and formamidinate bridging ligands (**3**–**6**); and the platinum-based drugs, cisplatin and carboplatin. The results reported in this work suggest that structural modifications of the dendritic molecules are required to improve their solubility in the biological medium. This would allow for accurate selectivity studies against healthy non-tumorigenic cell lines.

## 2. Results and Discussion

### 2.1. Synthesis and Characterization

**Figure 1:**
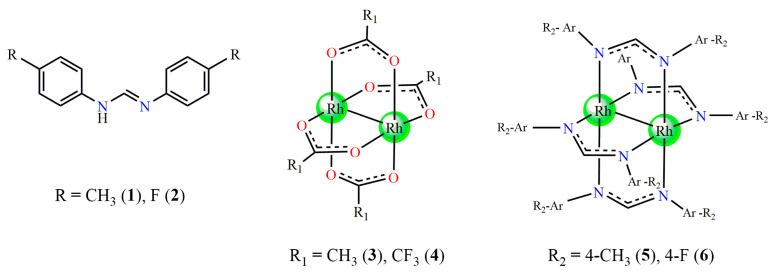
Structures of the ligands and dirhodium(II, II) complexes (**1**–**6**) [29].

The ligands (**1**, **2**) and bimetallic dirhodium(II,II) complexes (**3**–**6**) used in this study (Figure 1), were synthesized following previously published methods [35].

**Scheme 1:**
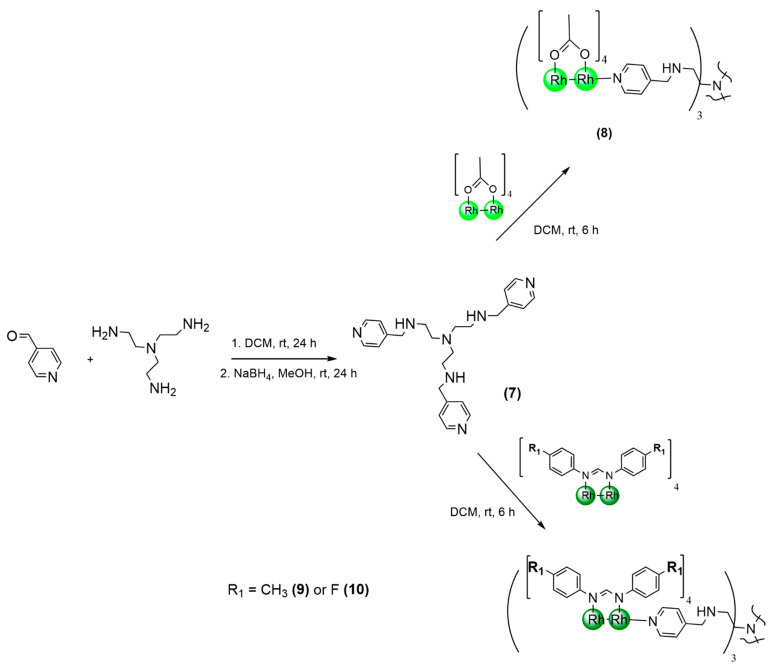
Synthetic method used to obtain low-valent dirhodium(II,II) metallodendrimers (**8**–**10**).

The known trivalent dendritic scaffold (**7**, Scheme 1) that is used to coordinate to the paddlewheel complexes was synthesized via a Schiff-base condensation reaction between 4-pyridinecarboxaldehyde and tris(2-aminoethyl)amine, which was then followed by a reduction in the imine functionality with sodium borohydride [36]. The new low-valent metallodendrimer compounds (**8**–**10**) evaluated in this study were obtained in good to excellent yields (76–84%). This was achieved by reacting the synthesized dirhodium(II,II) complexes (3.05–3.38 equivalents) with 1 equivalent of the pyridyl dendritic scaffold (**7**) in dichloromethane at ambient temperature (20–25 °C) for 6 h (Scheme 1). Single axial site functionalization was facilitated under stoichiometric control in order to achieve binding between the pyridyl nitrogen and dirhodium core. The acetate and formamidinate compounds (**8**–**10**) were found to be air stable and sparingly soluble in DCM, chloroform, acetone, and DMSO.

**Figure 2:**
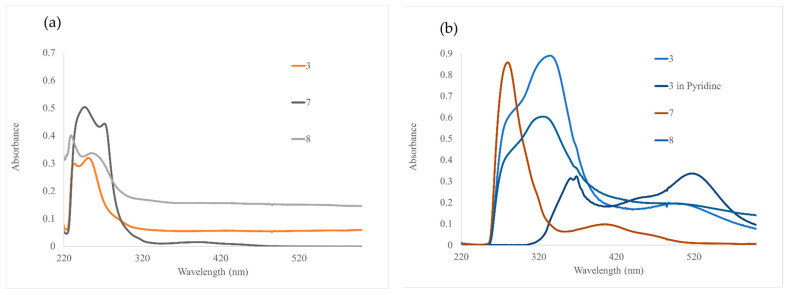
Electronic absorption spectra obtained for **3**, **7**, and **8** in (**a**) DCM and (**b**) DMSO. Complex **3** in pyridine is shown for comparison in (**b**).

The observed color changes in the reaction solution were ascribed to the axial site interaction of donor molecules with the dirhodium core unit, thus prompting an electronic absorption spectral analysis of compounds **3**, **7**, and **8** in order to corroborate the axial site binding of the scaffold (**7**). The electronic spectra were collected in coordinating (pyridine, DMSO) and non-coordinating (DCM) solvents for comparison. The distinctive absorption maxima of both precursor compounds (**3** and **7**) were noted. They were noted to differ from the absorbances observed in the spectrum obtained for complex **8**, which was recorded in DCM (Figure 2), thereby confirming the presence of a new species.

A similar trend in the data that were obtained for the same compounds analyzed in the coordinating solvent (Figure 2) was observed. It suggested a negligible displacement of the pyridyl moieties in the presence of DMSO. The spectrum obtained from complex **3** in pyridine shows further distinctive bands at 350, 360, and 520 nm due to the speculated *bis*-axial coordination of pyridine.

**Figure 3:**
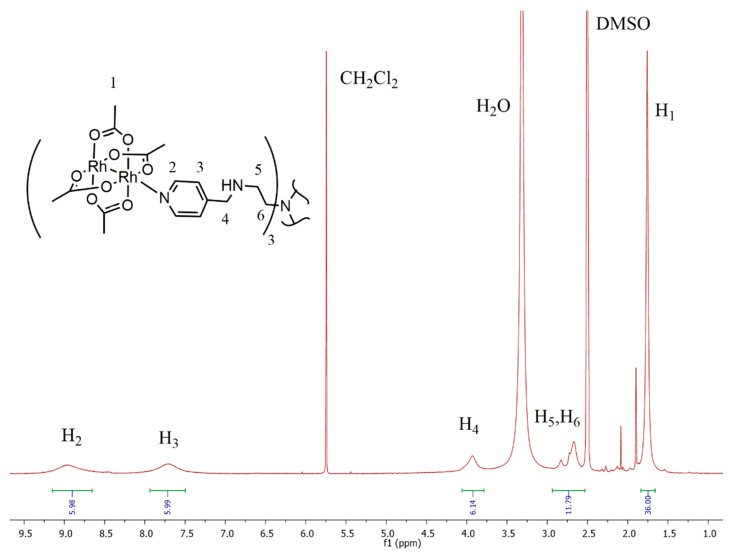
^1^H-NMR spectra obtained for complex **8** in DMSO-*d_6_*.

In the NMR spectra collected for **8**, a broadening and coalescing of certain resonance signals was observed. This feature was attributed to the high nuclearity of the dendritic complexes, as well as to the mobility of the dendritic arms with more rotational degrees of freedom, thereby leading to an averaging of signals on the NMR timescale. Additionally, complexes **8** and **9** exhibited poor solubility in the deuterated solvents, requiring a doping of the NMR samples with dichloromethane in order to produce sufficiently resolved spectra, thus further contributing to a lowered resolution of the observed signals for complex **8**. A low temperature NMR could not be attained to investigate the nature of the signals due to the observed precipitation of complex **8** from the solution and fusion temperature of DMSO. Nevertheless, the obtained ^1^H-NMR spectrum of acetate-containing complex **8** (Figure 3) displayed broadened signals that were observed at 9.01 ppm and corresponding to the protons adjacent to the pyridyl nitrogen atom. This was observed to shift from 8.47 ppm, as was previously observed in the ligand precursor **7**, thereby implicating the binding between the rhodium atom and the pyridyl nitrogen.

**Figure 4:**
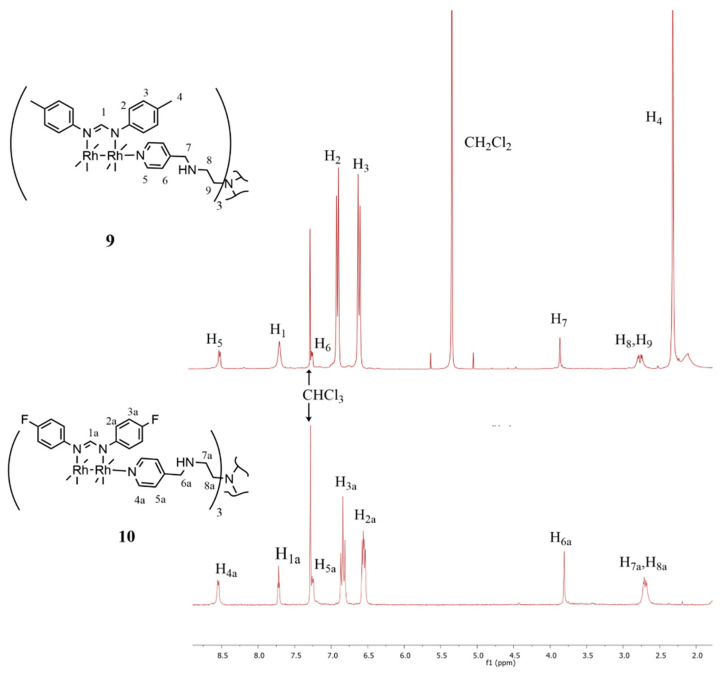
The ^1^H-NMR spectra obtained for **9** (above, DCM additive) and **10** (below) in CDCl_3_.

**Figure 5:**
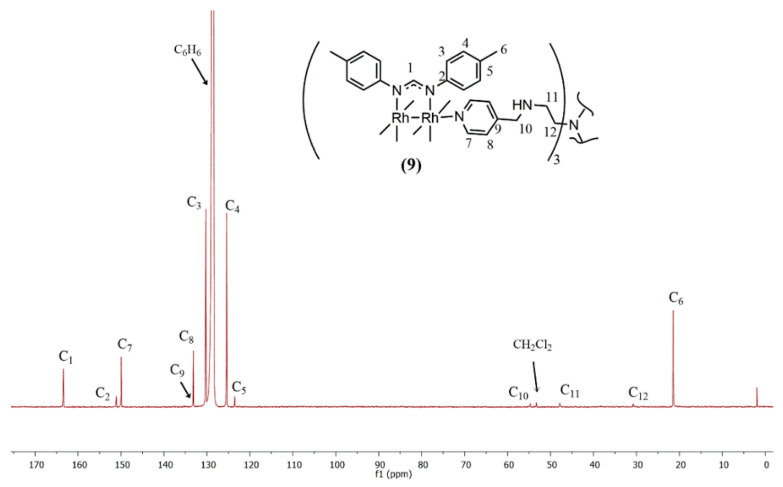
The ^13^C-NMR spectrum obtained for **9** in benzene-*d_6_*.

The formamidinate complexes **9** and **10** show an enhanced solubility in comparison to complex **8**; however, precipitation does occur over an extended time period in deuterated solvents. Despite the precipitation, the expected number of the signals and integration that was observed in the ^1^H NMR spectra for compounds **9** and **10** of the dendritic core was relative to the aromatic formamidinate signals. As in compound 8, downfield shifts are observed in the pyridyl doublet signals, supporting the mono-axial site functionalization of the proposed compounds **9** and **10** (Figure 4). The lack of an observed coalescence and broadening of the signals in the spectra of formamidinate complexes (**9**, **10**), relative to those observed for acetate compound **8**, was attributed to the steric influence of the ligated diphenylformamidinate moeities that was present on the conformational arrangements of the dendritic arms, which was coupled with the enhanced solubility exhibited by complexes **9** and **10**. In spite of the challenges regarding solubility, a ^13^C-NMR spectrum could be obtained for **9** in benzene-*d_6_*, and displays the expected signals pertaining to the interior scaffold and terminally bound dirhodium(II,II) units (Figure 5).

Additionally, the infrared spectra obtained for complexes **8**–**10** shows a shift in the pyridyl C=N absorption band from the 1610 cm^−1^ to 1586 cm^−1^ region, further supporting the coordination of the pyridyl nitrogen atom to the dirhodium core in each case (Figures S1–S3, Supplementary Materials). The weak band observed in the 3400 cm^−1^ regions in the IR spectra was ascribed to the secondary amine N-H stretching mode, thereby supporting a selective binding of the pyridyl nitrogen atom over the amine moiety. The positive-ion high resolution mass spectrometry (HR-ESI-MS) for complexes **8**–**10** reveals the base peaks corresponding at *m/z =* 1768.4221, *m/z* = 2072.4199, and *m/z* = 1294.4554, thereby corresponding to the [M + Na]^+^, ([M + 4(CH_2_Cl_2_) + 2K]^2+^) and ([M + 3Na]^3+^) ions for complexes **8**, **9**, and **10**, respectively. The complex fragmentation patterns observed in the mass spectra of complexes **8** and **10** were attributed to the macromolecular monodispersed nature of the dendritic structures, allowing for multiple points of fragmentation upon ionization (Figures S4–S6). Meanwhile, the low signal-to-noise ratio observed for **10** was attributed to the low solubility of the compound upon ESI analysis.

The encapsulation of solvent molecules within the structure that were seen for complex **9** has previously been reported for dendrimers [37,38]. The speculated encapsulation of the solvent was confirmed by a thermogravimetric analysis of complex **9** (Figure S7), whereby a percentage loss of 1.453% was observed between 32 and 110 °C, thus corresponding to the loss of one encapsulated DCM molecule. The loss at 120 °C may be attributed to conformational rotations of the dendritic arms, which results in the liberation of the remaining DCM molecules. The accompanying color change observed at this temperature was ascribed to a certain degree of decomposition, which was taking place until full decomposition occurred at around 400 °C.

The stability of the compounds in solution was evaluated by UV-Vis spectroscopy in DMSO at an ambient temperature (20–25 °C) over a 48 h period. No observable change in the electronic spectra (Figure S8) was noted for complex **8** in DMSO, thus supporting the spectral data and the stability of the Rh-N_pyridyl_ bond under these conditions. Although changes in the spectra for complexes **9** and **10** were observed, the comparison of these to the precursor complex spectra suggest a DMSO adduct formation in the first 2 h, followed by a stable species that was formed over the remainder of the elapsed time in the study (Figures S9 and S10).

### 2.2. In Vitro Cytotoxicity Measurements

The synthesized compounds were evaluated for their in vitro cytotoxicity against A2780, as well as in cisplatin-resistant (A2780*cis*) and OVCAR-3 ovarian cancer cell lines (Table 1). The cytotoxicity was determined using an MTT assay over a concentration range of 0.1–100 µM, as previously described [39]. Serial dilutions were carried out in such a way as to obtain the final concentration of compounds over the 0.1–100 µM concentration range with corresponding DMSO concentrations of 0.016–1%. Exceeding the maximal DMSO content of 1% is unfavorable, since higher DMSO concentrations may have an impact on the cell survival and influence that the IC_50_ values possess for the compounds [40]. Cisplatin and carboplatin were tested in parallel in the same concentration range for both ligands (**1**, **2**), dirhodium complexes (**3**–**6**), and metallodendrimers (**8**–**10**). These results are presented in Table 1 and Table S1. The sigmoidal dose–response curves (Supplementary Materials, Figures S11–S13) depicting the relationship between the survival rate and the concentration of the compounds yielded half maximal inhibitory concentrations or IC_50_ values (95% confidence interval). The solubility of the compounds did not allow a testing of over 100 µM, and the calculation of the quantitative IC_50_ value was not possible for the selected compounds. The testing of **7** was not carried out due to a negligible solubility in the medium, which is required for biological evaluation. Nonetheless, the absorbance values and, hence, the cell survival showed a continuous leaning decrease. Therefore, the linear regression was applied in order to analyze the dose–effect relationship. Furthermore, the statistical significance (*p* value < 0.05) of the calculated hillslope clearly demonstrated a cell growth inhibitory tendency in the studied concentration range; this was observed for all compounds in the A2780 cell line (Supplementary Materials, Figure S14, Table S1).

The compounds show a similar outcome in A2780*cis* and OVCAR-3 cell lines, with few exceptions: no changes were caused by the application of ligand **1** and complex **9** to the A2780*cis* and OVCAR-3 cell lines, nor by ligand **2** and complex **10** in the OVCAR-3 cell line. A pronounced increase in potency toward the A2780 cell line was observed for **2** with an IC_50_ value of 43.68 µM, although this result was approximately 6 times lower when compared to that of cisplatin (7.60 µM). Ligand **1** showed no appreciable activity in the tested concentration range in the A2780 cell line, and the IC_50_ values for ligands **1** and **2** in the A2780*cis* and OVCAR-3 cell lines could not be determined in the tested concentration range of 0.1–100 µM, which serves as a confirmation by the linear regression (see Table S1).

Inhibitory effects of the acetate-containing complexes (**3** and **4**)—with IC_50_ values of 45.65 and 52.57 µM, respectively—were found to favor the methyl-substituted complex (**3**). Interestingly, the application of complex **3** to the A2780*cis* cell line shows a marginally enhanced activity (43.25 µM) when compared to the A2780 cell line, as well as showed a greater cytotoxicity when compared to cisplatin (80.49 µM) or carboplatin (89.37 µM). Complex **4** showed no appreciable activity in the A2780*cis* cell line at the tested concentration range; however, its cytotoxicity was comparable to that of cisplatin, which was observed in the OVCAR-3 cell line. These results agree with previous literature findings, in which the interaction between dirhodium(II,II) complexes and DNA may allow for a growth inhibition through multiple pathways [32,33].

Complex **5** shows the greatest inhibitory effects of all tested compounds against the A2780, A2780*cis*, and OVCAR-3 cell lines with IC_50_ values of 36.18, 36.24, and 27.71 µM, respectively, thus exceeding cisplatin in all but the A2780 cell line. Notably, complex **5** was more active in the A2780, A2780*cis*, and OVCAR-3 cell lines than was observed for carboplatin, which is a commonly used drug in the treatment regime for ovarian cancer. Epithelial ovarian cancers such as OVCAR-3 are fast growing, spread throughout the peritoneal cavity and account for 90% of ovarian cancer diagnoses [41]. In this regard, the increased potency demonstrated by complex **5** in the OVCAR-3 cell line is encouraging. A substantial increase in efficacy across all cell lines when compared to the precursor ligand **1** was also observed, thereby suggesting a strong positive influence of the bimetallic center in this case. Complex **6** showed an increased cytotoxicity when compared to the precursor ligand **2** in the A2780 and OVCAR-3 cell lines, but an overall lower activity when compared to cisplatin. Generally, the coordination of complexes **3**–**6** to the tripyridyl scaffold **7** results in a lower cell growth inhibition, which is due to the previously observed solubility characteristics in the medium that are required for cytotoxic evaluation. Growth inhibition was observed in the application of the methyl-substituted compounds **8** and **10** in the A2780 cell line with observed IC_50_ values of 64.82 and 97.11 µM, respectively. This indicated that the terminally bound dirhodium(II,II) acetate moiety is a more potent agent than dirhodium(II,II) formamidinate analogs. Complex **8** shows the greatest cytotoxicity of the metallodendrimers in the A2780 and OVCAR-3 cell lines with IC_50_ values of 64.82 and 69.05, respectively. This suggests a positive antiproliferative effect in the OVCAR-3 cell line by binding complex **3** to the termini of the dendritic scaffold, which was not seen for complexes **9** and **10**. This may be due to a mechanism of action where an additive effect of the suitable physico-chemical properties of the acetate-bearing compound **8** results in enhanced activity when compared to complex **3**.

## 3. Materials and Methods

### 3.1. General Information

All chemicals and reagents were purchased from Merck Chemicals (Darmstadt, Germany) and used as is, unless otherwise stated. The precursor metal salt, Rhodium(III) trichloride trihydrate, was obtained from Heraeus (Gqeberha, South Africa). Solvents used in synthesis and the deuterated solvents for analysis were freshly distilled and stored over molecular sieves. All reactions were carried out using standard Schlenk techniques under an inert (N_2_) atmosphere. The ligands (**1**, **2**) [1], complexes (**3**–**6**) [35], and tripyridyl scaffold (**7**) [36] were synthesized according to the published literature procedures.

### 3.2. Instruments

Nuclear magnetic resonance (NMR) spectra were recorded on a Bruker (Billerica, MA, USA) X400 (^1^H: 400.22 MHz, ^13^C{^1^H}: 100.65 MHz) or Varian (Palo Alto, CA, USA) Mercury 300 (^1^H: 300.08 MHz, ^13^C{^1^H}: 75.46 MHz) spectrometer at a temperature of 298 K. Tetramethylsilane (TMS) was used as the internal standard for referencing chemical shifts (in ppm). The coupling constants were reported in Hz. Infrared (IR) spectra and were obtained from a Perkin-Elmer (Waltham, MA, USA) Spectrum 100 FT-IR spectrometer using attenuated total reflectance infrared spectroscopy (ATR-IR). The mass spectrometric data were obtained using Electron Impact (EI) on a JEOL (Akishima, Tokyo, Japan) GC matell instrument, or via using a Waters (Milford, MA, USA) Synapt G2, which was equipped with an ESI probe with data recorded in positive mode. Electronic absorption spectra were recorded on an Agilent (Santa Clara, CA, USA) Cary 8454 Photodiode Array UV/Visible spectrometer. Elemental analyses (C, H, N) were performed using a Perkin Elmer (Waltham, MA, USA) 2400 II (CHN/S) Analyzer. The melting points were obtained on a Büchi (Meierseggstrasse, Switzerland) B-540 melting point apparatus and were reported without correction. The thermogravimetric analysis (TGA) measurements were carried out on a Waters (Milford, MA, USA) TGA Q500 instrument, utilizing a heating rate of 10 °C min^−1^ under a dry nitrogen flow of 60 mL min^−1^. The biologic testing was performed in a cell culture laboratory equipped with Class II laminary hoods (Streamline from Esco, Changi, Singapore); a cell culture incubator (Revco RGT-5000T-9-VBC, Thermo Electron Corporation, Asheville, NC, USA); a centrifuge with a swing-out rotor (32 R from Hettich Lab Technology, Tuttlingen, Germany); an inverted phase microscope (CKX41 Olympus, Tokio, Japan); an automatic cell counter (Eve NanoEnTek, Seoul, Republic of Korea); a microplate reader with a monocromator (Synergy 2.0 from BioTek Company, Winooski, VT, USA); an orbital shaker (PSU-10i from BioSan, Riga, Latvia); and a Titramax1000 shaking platform with incubation capacities (Heidolph Instruments GmbH, Schwabach, Germany).

### 3.3. Synthetic Procedure for Trimeric Dirhodium(II,II) Metallodendrimers

The general method for the synthesis of trimeric dirhodium(II,II) metallodendrimers was carried out as follows: A round-bottomed flask equipped with a magnetic stirrer bar was charged with a suspension of either compound **3** (0.050 g, 0.11 mmol, 3.29 eq.), or a solution of either compound **5** (0.040 g, 0.036 mmol, 3.05 eq.) or compound **6** (0.050 g, 0.0440 mmol, 3.38 eq.) in dry dichloromethane (10 mL) under an inert atmosphere. A solution of **7** (0.015 g, 0.340 mmol, 1.00 eq.) in dry dichloromethane (5 mL) was added dropwise and the reaction was stirred at ambient temperature for 6 h. The reaction mixture was concentrated to ca. 5 mL under reduced pressure. Petroleum ether (10 mL) was added to the concentrated reaction mixture with a subsequent formation of red (**8**) or dark green (**10**, **11**) precipitates. The solid was filtered under a vacuum and washed with petroleum ether (5 × 10 mL). The products were dried under vacuum, either affording red (**8**), crystalline solid, or green (**9**, **10**) crystalline powders.

#### 3.3.1. Tris-(dirhodium(II,II) tetraacetate) Metallodendrimer (**8**)

Yield: 0.052 g, 80%. Melting point: 352.4 °C (decomposition without melting). FTIR (υ, cm^−1^) = 3425 (m, N-H), 1586 (s, C=N_pyridyl_), 1545 (s, C=O), 1440 (m, C-O). ^1^H NMR (300 MHz, DMSO-*d_6_*): δ (ppm) = 9.01 (br s, 6H, H_1a_), 7.72 (br s, 6H, H_2a_), 3.89 (br s, 6H, H_3a_), 2.85 (br s, 12H, H_4a_, H_5a_), 1.76 (s, 36H, H_6a_). MS(HR-ESI)(*m*/*z*): 1768.4221 (100%, [M + Na]^+^, calcd. 1768.5300). Elemental analysis for C_48_H_69_N_7_O_24_Rh_6_·3H_2_O·DCM: Found C, 30.87, H, 4.24, N, 5.08%; calcd. C, 31.21, H, 4.17, N, 5.20%.

#### 3.3.2. Tris-(dirhodium(II,II) tetrakis(di-*p*-tolylformamidinate)) Metallodendrimer (**9**)

Yield: 0.051 g, 76%. Melting point: Darkening onset at 120 °C, full decomposition without melting at 400 °C. FTIR (υ, cm^−1^) = 3421 (m, N-H), 1592 (s, C=N_pyridyl_), 1583 (s, C=N_formamidinate_). ^1^H NMR (300 MHz, Benzene-*d_6_*): δ (ppm) = 8.55 (d, *^3^J =* 5.9 Hz, 6H, H_7b_), 7.81 (t, *^3^J =* 3.3 Hz, 12H, H_1b_), 6.91 (d, *^3^J =* 5.9 Hz, 6H, H_8b_), 6.83 (dd, *^3^J* = 20.8, 8.3 Hz, 96H, H_3b_, H_4b_), 3.35 (s, 6H, H_10b_), 2.30 (m, 12H, H_11b_, H_12b_), 2.07 (s, 72H, H_6b_). ^13^C{^1^H}NMR (151 MHz, Benzene-*d_6_*): δ (ppm) = 163.4 (s, C_1b_), 151.1 (s, C_2b_), 149.9 (s, C_7b_), 133.3 (s, C_9b_), 133.1 (s, C_8b_), 130.3 (s, C_3b_), 125.4 (s, C_4b_), 123.5 (s, C_5b_), 54.8 (s, C_10b_), 47.8 (s, C_11b_), 31.5 (s, C_12b_), 21.4 (s, C_6b_). MS(HR-ESI)(*m*/*z*): 2072.4119, (100%, [M + 4(CH_2_Cl_2_) + 2K]^2+^, calcd. 2072.4528). Elemental analysis for C_204_H_213_N_31_Rh_6_·DCM: Found C, 64.65, H, 5.94, N, 10.95%; calcd. C, 64.79, H, 5.68, N, 11.19%.

#### 3.3.3. Tris-(dirhodium(II,II) tetrakis(bis-(4-fluorophenyl)formamidinate)) Metallodendrimer (**10**)

Yield: 0.046 g, 84%. Melting point: 397.7–398.5 °C. FTIR (υ, cm^−1^) =3267 (m, N-H), 1590 (s, C=N_pyridyl_), 1581 (s, C=N_formamidinate_), 1195 (s, C-F). ^1^H NMR (300 MHz, DMSO-*d_6_*): δ (ppm) = 8.52 (d, *^3^J =* 5.9 Hz, 6H, H_6c_), 7.68 (t, *^3^J =* 2.8 Hz, 12H, H_1c_), 7.24 (d, *^3^J =* 5.8 Hz, 6H, H_7c_), 6.82 (t, *^3^J =* 6.9 Hz, 48H, H_4c_), 6.55 (dd, *^3^J =* 6.9 Hz, 3.7 Hz, 48H, H_3c_), 3.69 (s, 6H, H_9c_), 2.68 (br s, 12H, H_10c_, H_11c_). MS(HR-ESI)(*m*/*z*): 1294.4554 (100%, [M + 3Na]^3+^, calcd. 1294.4862). Elemental analysis for C_180_H_141_F_24_N_31_Rh_6_·3H_2_O: Found C, 55.96, H, 3.72, N, 10.78%; calcd. C, 55.93, H, 3.83, N, 11.05%.

### 3.4. Cell Growth Inhibition

The in vitro study was performed on three ovarian cell lines: the A2780 platinum sensitive ovary carcinoma, the A2780*cis* cisplatin-resistant ovary carcinoma, and the OVCAR-3 adenocarcinoma cell lines. A2780 and A2780*cis* cells were acquired from the European Collection of Authenticated Cell Cultures through Sigma Aldrich, St. Louis, MO, USA, while the OVCAR-3 cell line was from American Type Culture Collection (Manasses, VA, USA, acquired through LGC Standards GmbH, Wesel, Germany). The A2780 and A2780-*cis* cells were cultured in RPMI-1640 cell culture media, which were supplemented with 10% fetal bovine serum; OVCAR-3 and 20% fetal bovine serum; and 0.01 mg/mL insulin from a porcine pancreas (all media and supplements were from Sigma Aldrich, St. Louis, MO, USA). For cytotoxicity testing, the cells were seeded on 96-well Nunclon Delta surface assay plates (from Thermo Scientific, Waltham, USA); in addition to in wells containing 190 µL cell suspensions. The compounds **1**–**6** and **8**–**10** were diluted in dimethyl sulfoxide (DMSO, from Merck, Darmstadt, Germany) to obtain 10 mM stock solutions, and serial dilutions were prepared using a 0.01M PBS solution in order to obtain concentrations in the range of 2–2000 µM. Cisplatin and carboplatin were received from Actavis, through Sindan-Pharma Srl, Bucharest, Romania. The platinum-based drugs were diluted in a physiological serum, and serial dilutions were made in PBS to obtain the same concentration range as for Rh_2_(II,II) compounds at 2–2000 µM. For testing, each well containing cells was treated with the respective compound in a proportion of 1:20 (compound: cell culture media) in order to obtain a final concentration range of 0.1–100 µM for the compounds in the cell culture media. In every well, a 10µL compound was pipetted and the plates were incubated for 24 h, in triplicate. Untreated cells were used as a reference for 100% cell growth. In addition, the cell culture media without cells were used as a blank, and wells filled with cell culture media and the serial dilutions of each compound were used as color control; three independent measurements were performed. To assess the cytotoxicity of the compounds, the colorimetric MTT (3-(4,5-dimethylthiazol-2-yl)-2,5-diphenyltetrazolium bromide) reduction assay was performed [39]. Briefly, the cell culture media was gently removed from the 96-well plates. The wells were filled with 100µL of 1mg/mL MTT solution (MTT reagent and its solvent, the Hank’s media were purchased from Sigma Aldrich) and the samples were incubated for 1 h. The MTT solution was removed and in each well 150 µL DMSO was dispensed. After a short (5–10 min) shaking on a shaking platform with incubation, the optical density of each well was measured at 570 nm on a microplate reader. Furthermore, the absorbance data were implemented using GraphPad Prism 5 software (from GraphPad Software, La Jolla, USA) to obtain nonlinear, sigmoidal curves, and IC50 values. Additionally, the linear regression of each dose–response dataset was also analyzed in the 95% confidence interval.

## 4. Conclusions

In summary, new trivalent metallodendrimers bearing dirhodium(II,II) complexes bound on the periphery were prepared in good yields (76–84%); they were characterized by various spectroscopic and analytical techniques. The dirhodium compounds **2**–**6** show a cytotoxicity that was greater than or comparable to carboplatin in the A2780 cell line; none of which, however, exceeded the cytotoxicity of cisplatin in this cell line. Compounds **3** and **5** showed an approximate two-fold increase in cytotoxicity in the A2780*cis* cell line when compared to cisplatin and carboplatin, whereas compounds **4** and **5** show the highest antiproliferative effect of the synthesized compounds in the OVCAR-3 cell line. Generally, the results for dendritic complexes (**8**–**10**) show a decrease in cytotoxicity when compared to the bimetallic precursor complexes, with acetate-containing metallodendrimer (**8**) displaying the greatest antiproliferative effect for the macromolecular series of compounds. Compound **5** shows promising cytotoxicity against highly proliferative human ovarian tumor cell lines in vitro, having a comparable to, or greater, activity in comparison to the tested platinum-based drugs, with similar efficacy observed across all tested cell lines. Further investigations are underway to improve on the design strategy and fine-tuning of the physicochemical properties toward the investigation of the mechanisms of action on which these complexes exert their influence, thus their selectivity for cancer cells over healthy cells may then be determined.

## Figures and Tables

**Table 1:** The cell growth inhibitory capacity of formamidinate ligands (**1**, **2**), dirhodium(II,II) complexes (**3**–**10**), as well as the carboplatin and cisplatin in cancer cell lines (A2780, A2780*cis*, and OVCAR-3) were all expressed as half-inhibitory concentrations IC_50_ (µM).

Compound	*n* ^a^	Sigmoidal Dose–Response IC_50_(µM) ^b^		
		A2780	A2780*cis*	OVCAR-3
Cisplatin	1	7.60 ± 1.34	80.49 ± 0.74	30.57 ± 0.85
Carboplatin	1	53.06 ± 2.52	89.37 ± 2.32	45.90 ± 0.91
**1**	0	*	*	*
**2**	0	43.68 ± 0.99	*	*
**3**	2	45.65 ± 0.97	43.25 ± 0.94	*
**4**	2	52.57 ± 0.81	*	32.22 ± 0.76
**5**	2	36.18 ± 0.97	36.24 ± 0.97	27.71 ± 1.09
**6**	2	41.17 ± 0.87	*	72.21 ± 0.74
**8**	6	64.82 ± 1.23	*	69.05 ± 0.52
**9**	6	*	*	*
**10**	6	97.11 ± 1.02	*	*

^a^ Number of metal atoms per molecule, ^b^ IC_50_ value ± standard error, * Exceeded the 100 µM testing limit.

## Data Availability

Data is contained within the available article and Supplementary Materials.

## Supplementary Materials

The following supporting information can be downloaded at: https://www.mdpi.com/article/10.3390/molecules28062671/s1, Figures S1–S3: Infrared spectra obtained for compounds **8**–**10**; Figures S4–S6: HR-ESI MS spectra for compounds **8**–**10**; Figure S7: TGA trace obtained for compound **9**. Figures S8–S10: Electronic spectra for compounds **8**–**10** in DMSO at t = 0, 2 and 48 h; Figure S11: Dose-response curves for **1**–**6**, **8**–**10** and cisplatin in the A2780 cell line; Figure S12: Dose-response curves for **1**–**6**, **8**–**10** and cisplatin in the A2780*cis* cell line; Figure S13: Dose-response curves for **1**–**6**, **8**–**10** and cisplatin in the OVCAR-3 cell line. Figure S14: Dose-response linear regression of optical density versus concentration, in ovarian tumor cells subjected to treatment for 24 h. Table S1: Cell growth inhibitory capacity of formamidinate ligands (**1**, **2**), dirhodium(II,II) complexes (**3**–**10**) and cisplatin in cancer cell lines.

## Sample Availability

Samples of the compounds **1**–**10** are not available from the authors.

## Abbreviations

DCM	Dichloromethane
DMSO	Dimethylsulfoxide
DNA	Deoxyribonucleic acid
HR-ESI-MS	High Resolution Electrospray Ionization Mass Spectrometry
MTT	3-(4,5-dimethylthiazol-2-yl)-2,5-diphenyltetrazolium bromide
NMR	Nuclear magnetic resonance
IC_50_	Concentration required for 50% cell growth inhibition
