# Supplementary material for: Cytotoxicity Evaluation of Unmodified Paddlewheel Dirhodium(II,II)-Acetate/-Formamidinate Complexes and Their Axially Modified Low-Valent Metallodendrimers
Source: Molecules. 2023 Mar 15;28(6):2671. doi: 10.3390/molecules28062671 (PMC10055960; doi:10.3390/molecules28062671)
Supplement: Supplementary file 1 [file molecules-28-02671-s001.zip › molecules-2223002-supplementary.pdf]

# SUPPLEMENTARY MATERIALS

# Cytotoxicity evaluation of unmodified paddlewheel dirhodium(II,II)-acetate/-formamidinate complexes and their axially modified low-valent metallodendrimers

Stephen de Doncker [1], Eva Fischer-Fodor [2], Cătălin Ioan Vlad [3,4],

Patriciu Achimas-Cadariu [3,4], Gregory S. Smith [1,]* and Siyabonga Ngubane [1,]*

[1] Department of Chemistry, University of Cape Town, Rondebosch, Cape Town 7701, South Africa
[2] Tumour Biology Department, The Oncology Institute "Prof. Dr. Ion Chiricuta", 400015 Cluj-Napoca, Romania
[3] Department of Surgery, The Oncology Institute, "Prof. Dr. Ion Chiricuţă", 400015 Cluj-Napoca, Romania
[4] Department of Surgery and Gynecological Oncology, "Iuliu Hatieganu" University of Medicine and Pharmacy, 400337 Cluj-Napoca, Romania

# Table of Contents

Content

Cover Page……………….……………………………………………………………...1

Table of contents…………………………………………………………………...……2

**Figure S1**. Infrared spectrum obtained for **8**..………….……..…………………………....3

**Figure S2**. Infrared spectrum obtained for **9**...………….……..…………………………....3

**Figure S3**. Infrared spectrum obtained for **10**..………….……..…………..………………4

**Figure S4**. HR-ESI-MS spectrum obtained for **8**……………...….……………………….4

**Figure S5**. HR-ESI-MS spectrum obtained for **9**……………...….……………………….5

**Figure S6**. HR-ESI-MS spectrum obtained for **10**……..……...….…………………….....5

**Figure S7**. TGA trace obtained for **9**..........................................…………………………....6

**Figure S8**. Electronic absorption spectra of **8** in DMSO collected at t = 0, 2 and 48 h...........6

**Figure S9**. Electronic absorption spectra of **9** in DMSO collected at t = 0, 2 and 48 h...........7

**Figure S10**. Electronic absorption spectra of **10** in DMSO collected at t = 0, 2 and 48 h ......7

**Figure S11**. Dose-response curves for **1-6**, **8-10** and cisplatin in the A2780 cell line............8

**Figure S12**. Dose-response curves for **1-6**, **8-10** and cisplatin in the A2780*cis* cell line.......8

**Figure S13**. Dose-response curves for **1-6**, **8-10** and cisplatin in the OVCAR-3 cell line......9

**Figure S14**. Dose-response linear regression of optical density *versus* concentration, in ovarian tumor cells subjected to treatment for 24 hours……………………………………..10

**Table S1**. The cell growth inhibitory capacity of formamidinate ligands (**1**, **2**), dirhodium(II,II) complexes (**3 - 10**) and cisplatin in cancer cell lines..................................11

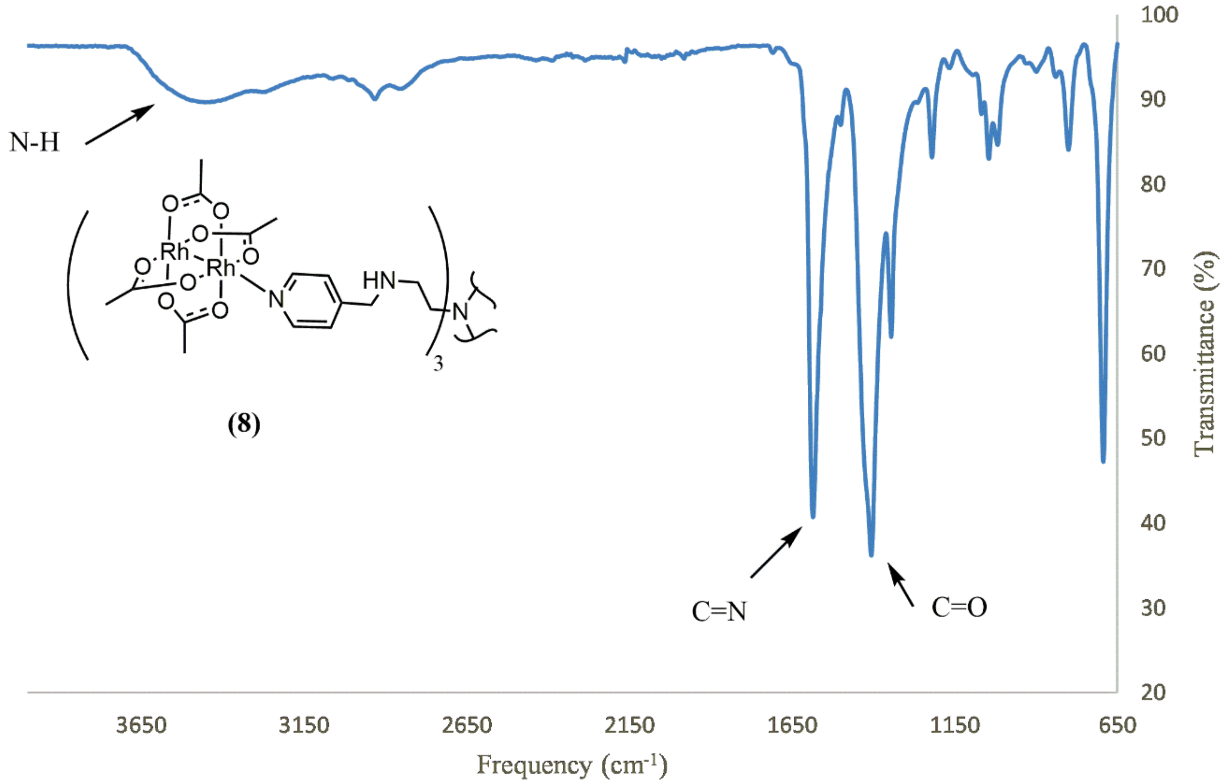


**Figure S1**. Infrared spectrum obtained for **8**

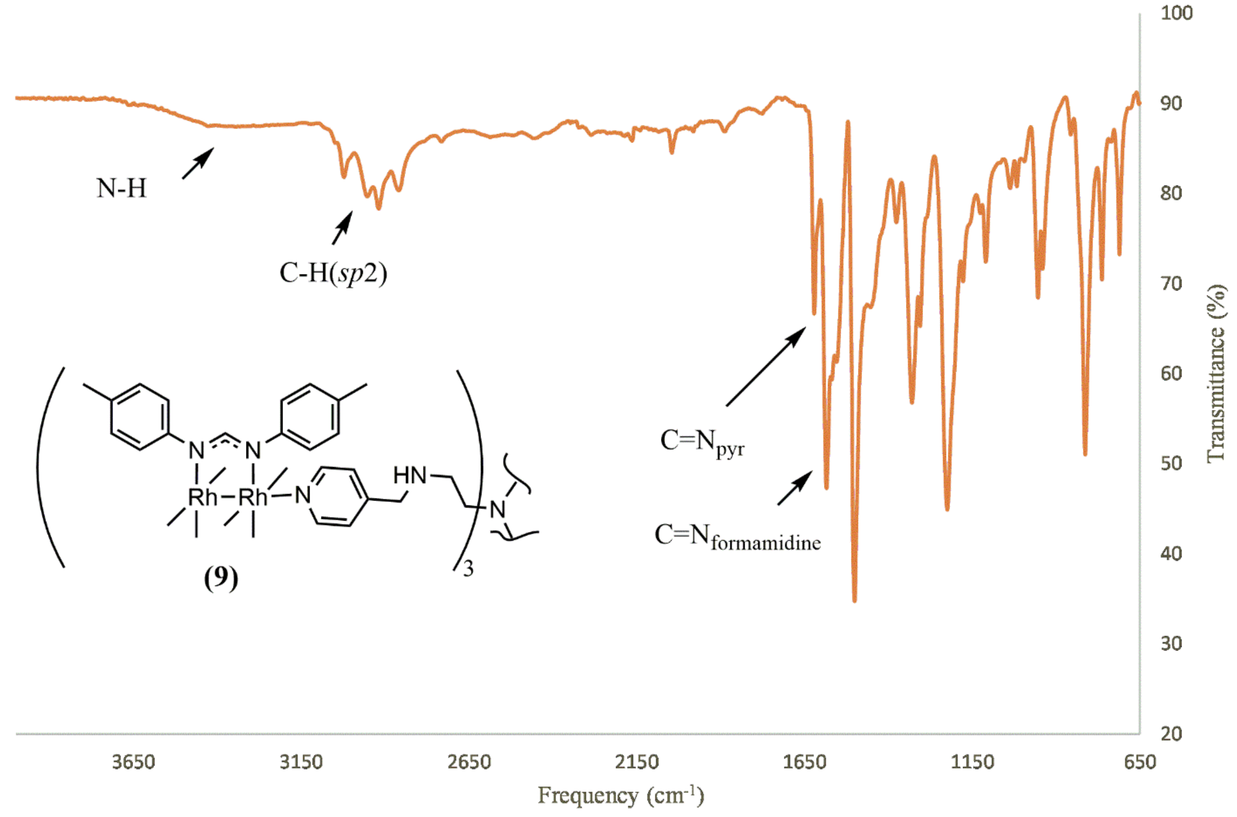


**Figure S2**. Infrared spectrum obtained for **9**.

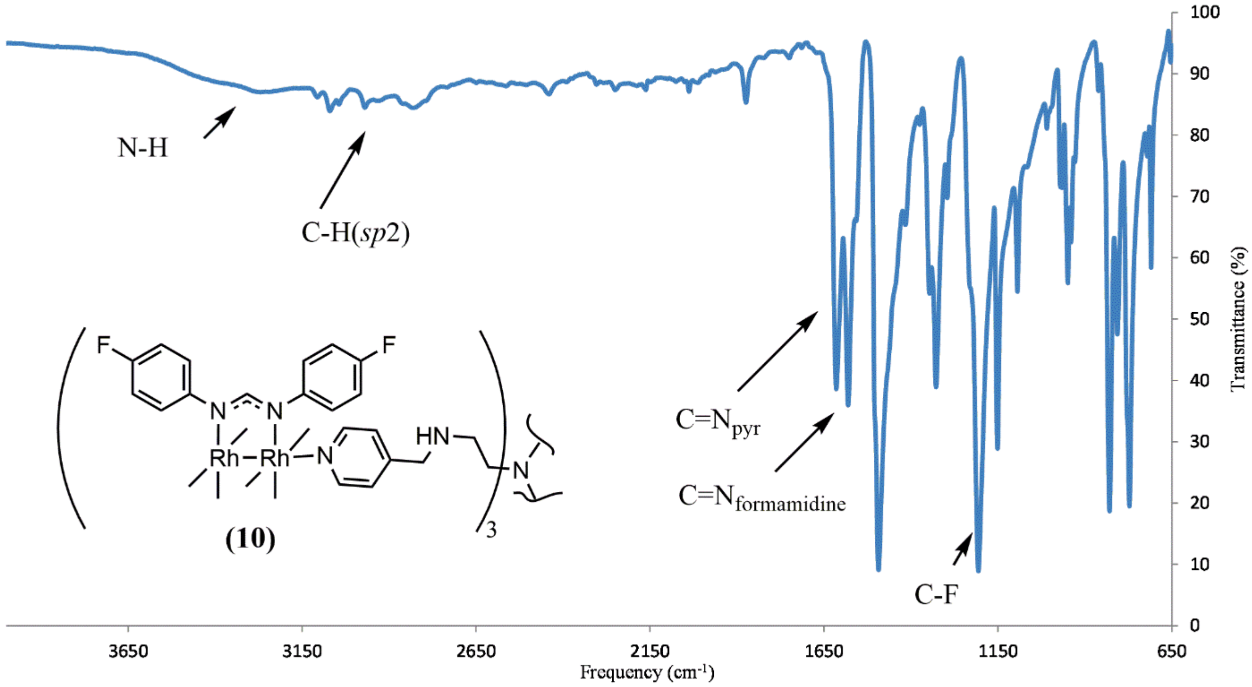


**Figure S3**. Infrared spectrum obtained for **10**.

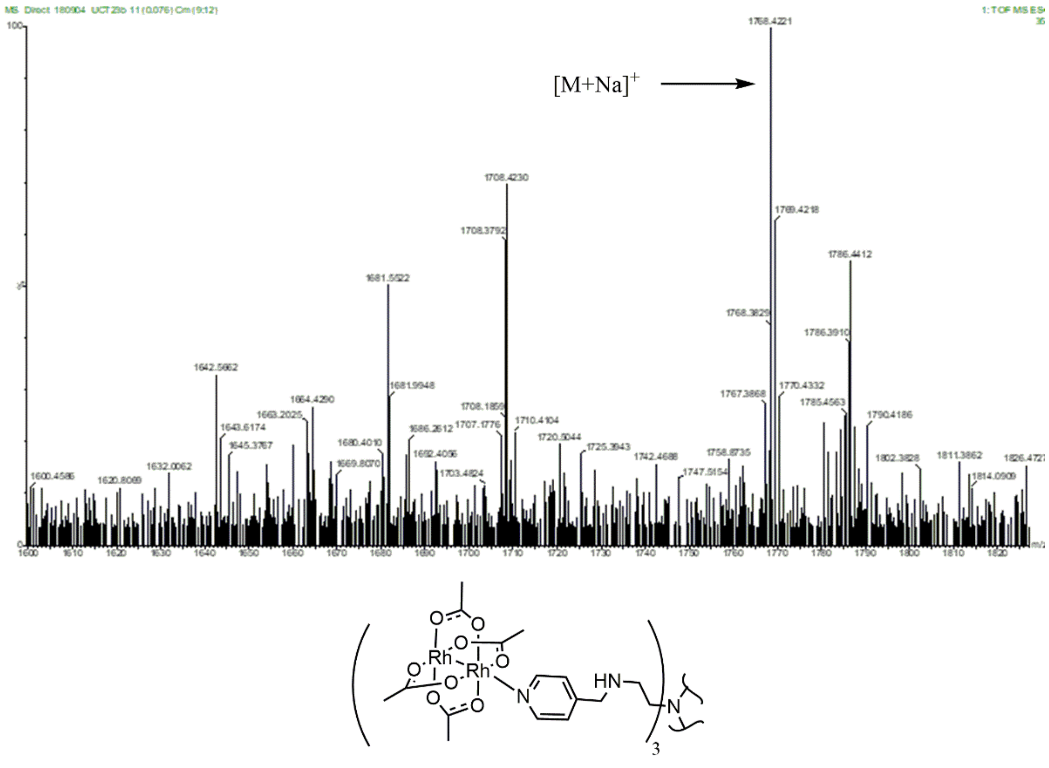


**(8)**

**Figure S4**. HR-ESI-MS spectrum obtained for **8**.

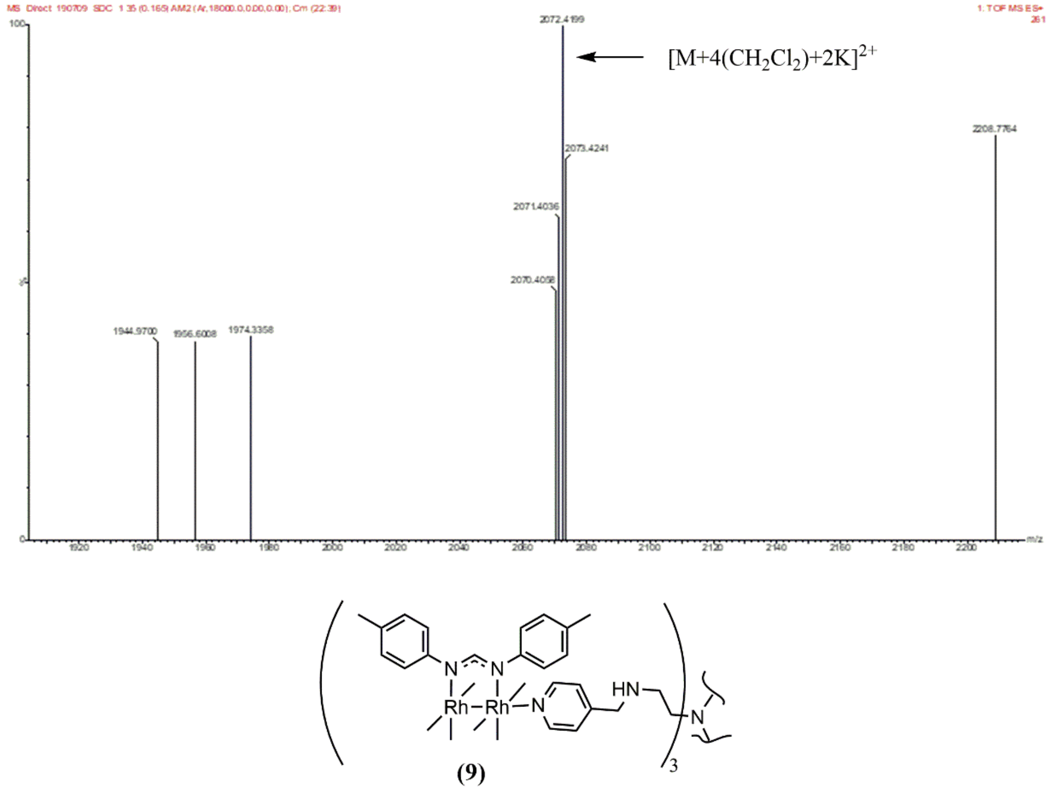


**Figure S5**. HR-ESI-MS spectrum obtained for **9**.

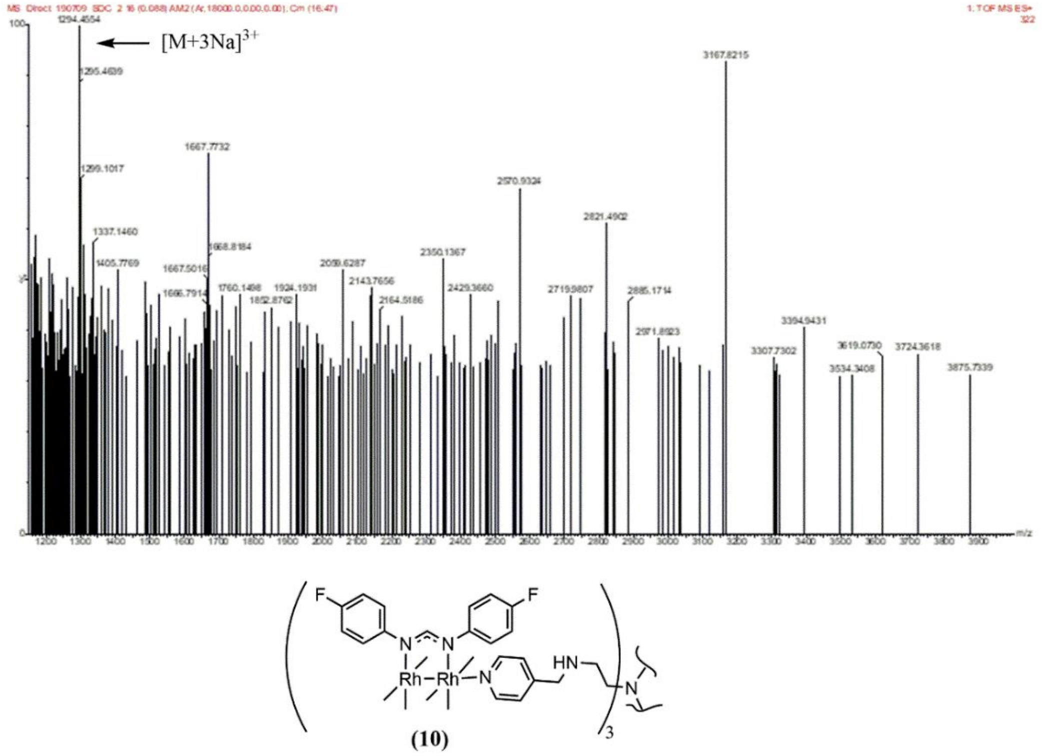


**Figure S6**. HR-ESI-MS spectrum obtained for **10**.

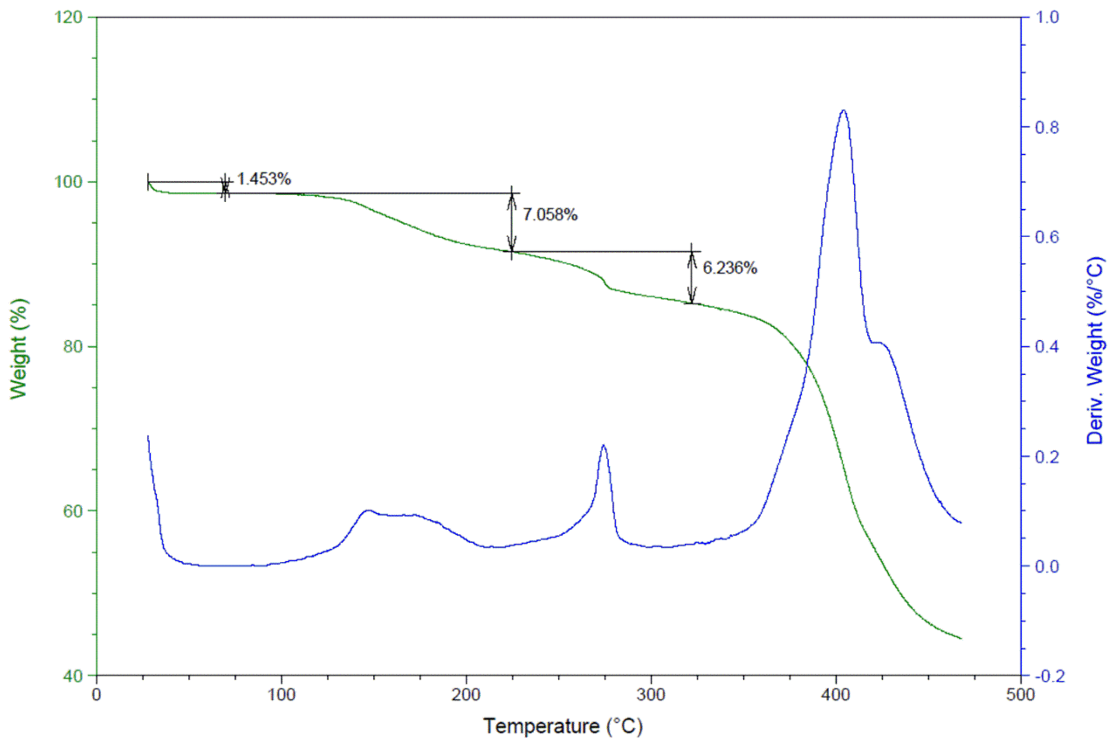


**Figure S7**. TGA trace obtained for compound **9**.

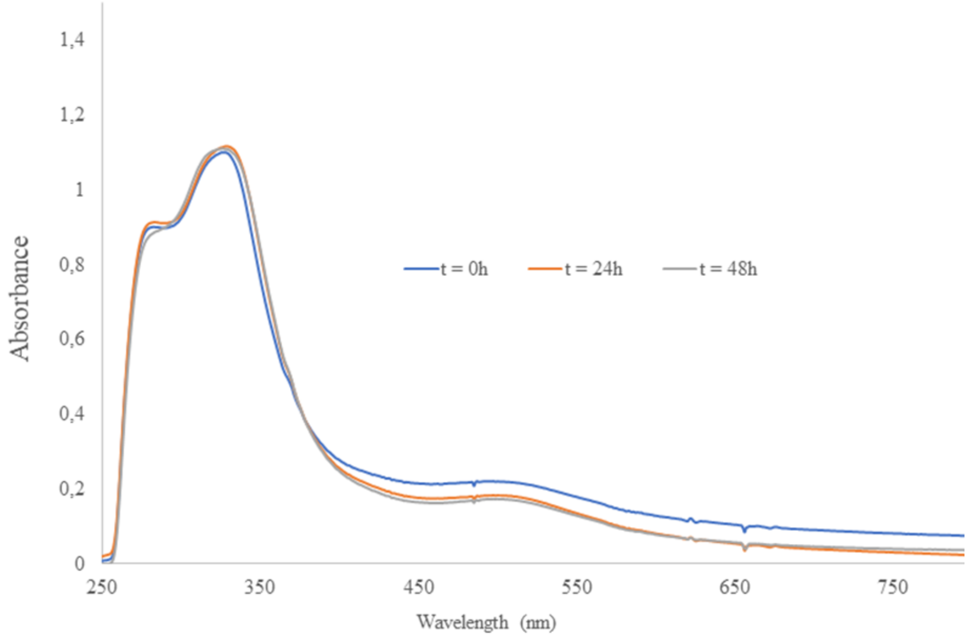


**Figure S8**. UV-vis stability of complex **8** in DMSO collected at t = 0, 2 and 48 h.

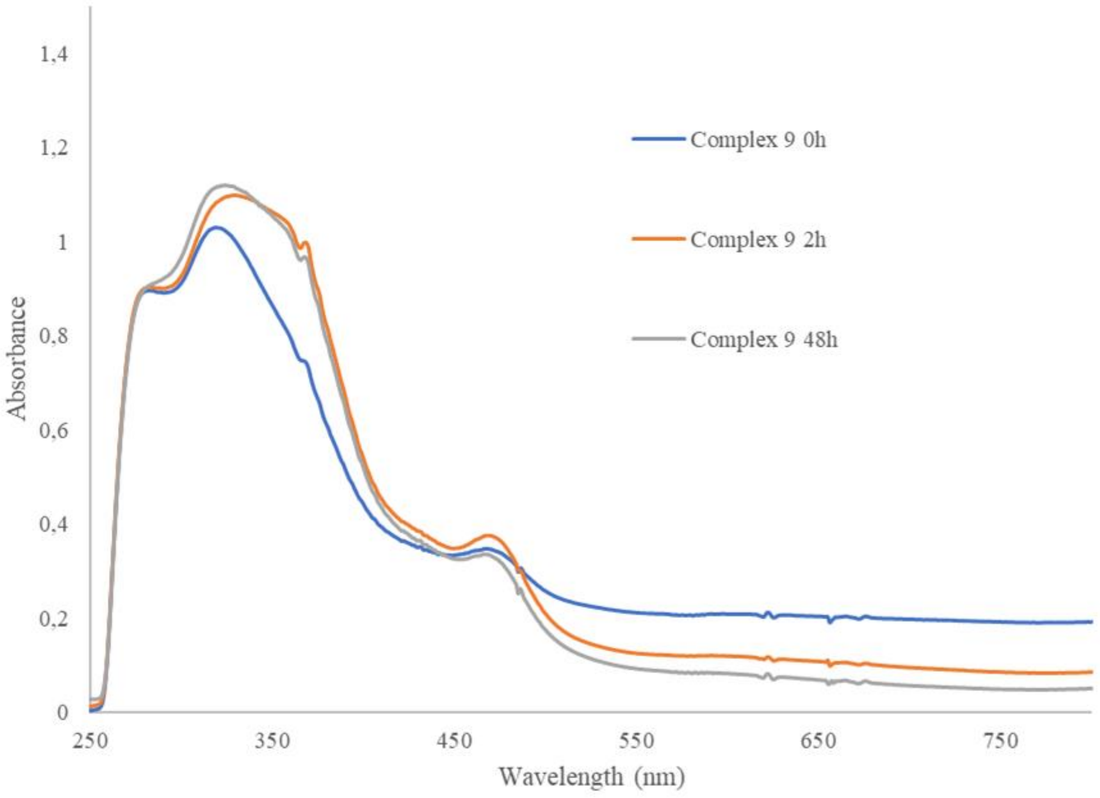


**Figure S9**. Electronic absorption spectra of **9** in DMSO collected at t = 0, 2 and 48 h.

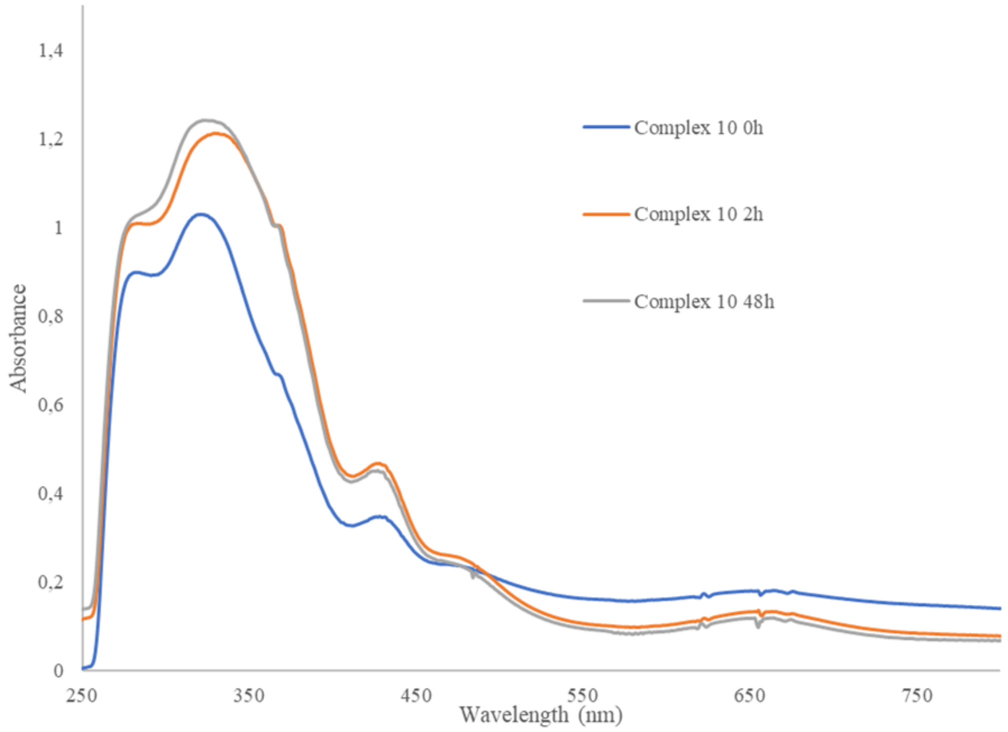


**Figure S10**. Electronic absorption spectra of **10** in DMSO collected at t = 0, 2 and 48 h.

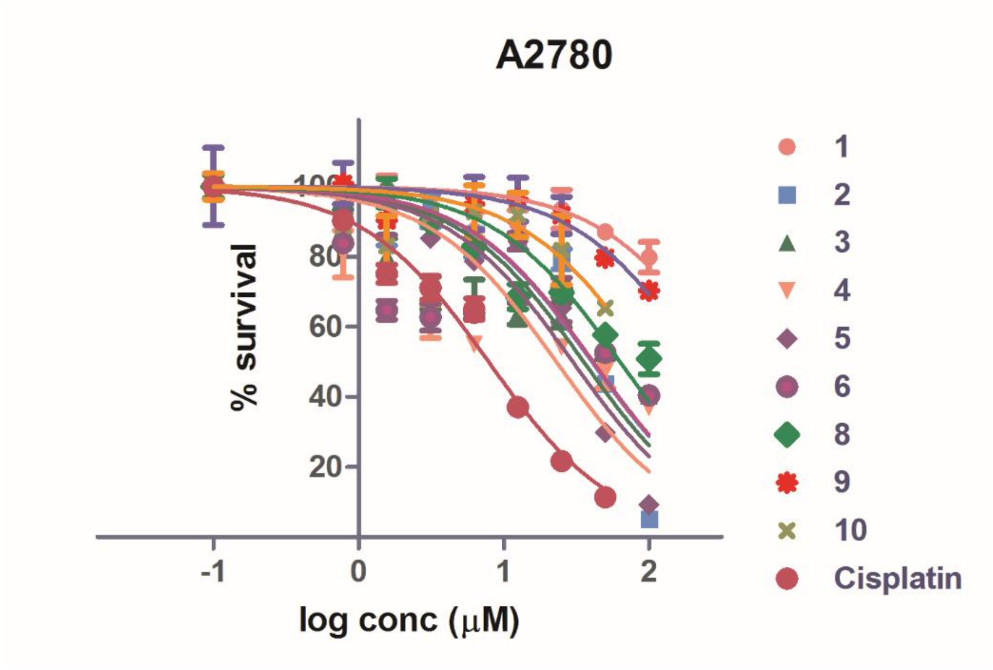


**Figure S11**. Dose-response curves for **1-6**, **8-10** and cisplatin in the A2780 cell line.

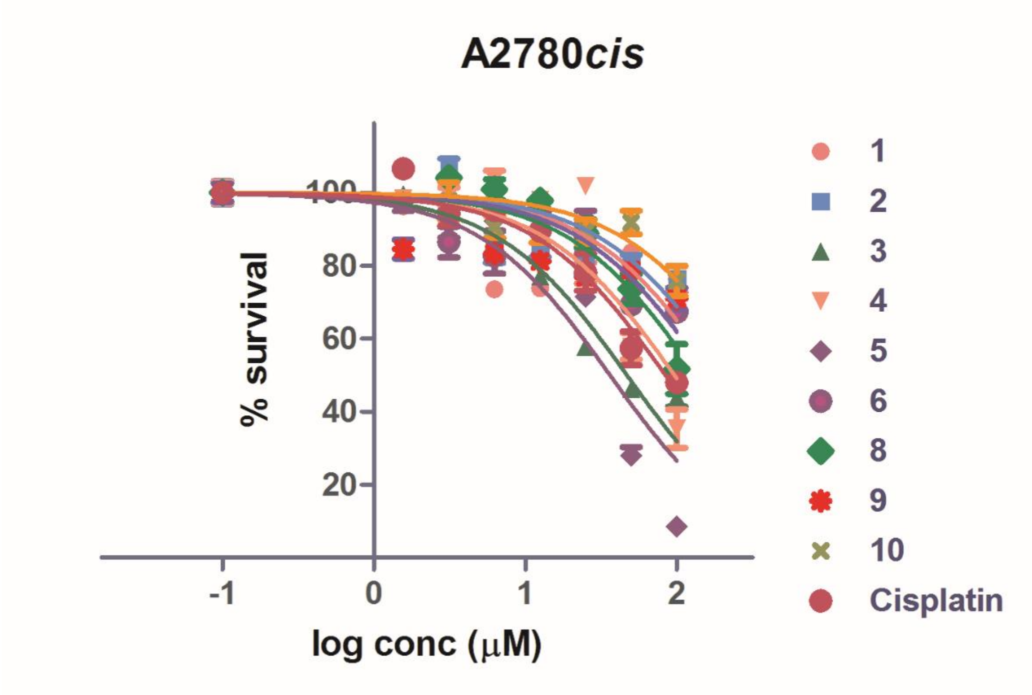


**Figure S12**. Dose-response curves for **1-6**, **8-10** and cisplatin in the A2780*cis* cell line.

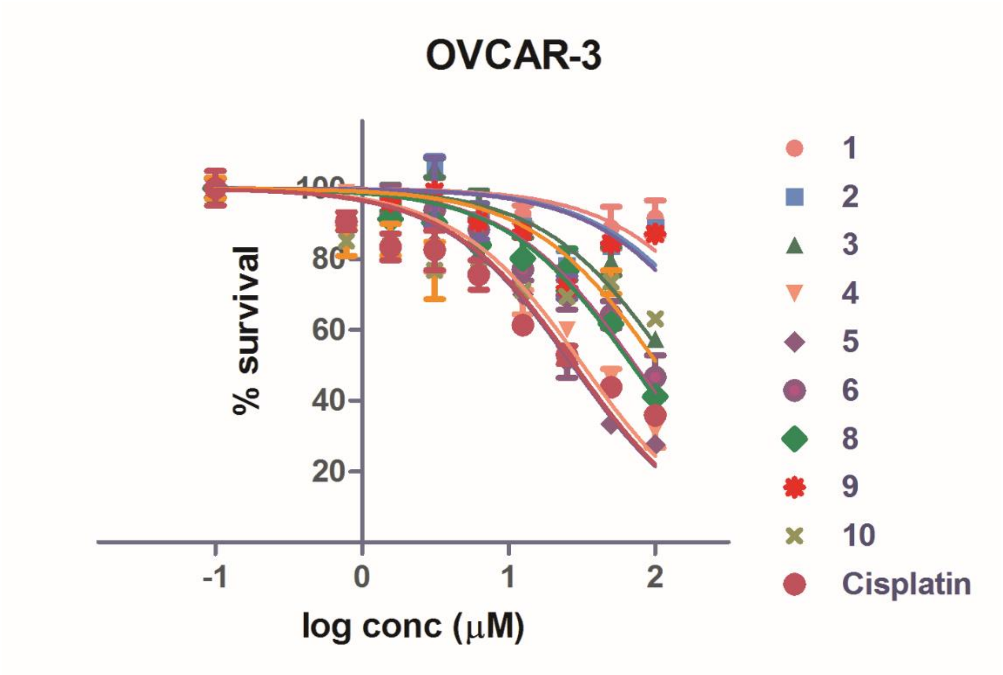


**Figure S13**. Dose-response curves for **1-6**, **8-10** and cisplatin in the OVCAR-3 cell line.

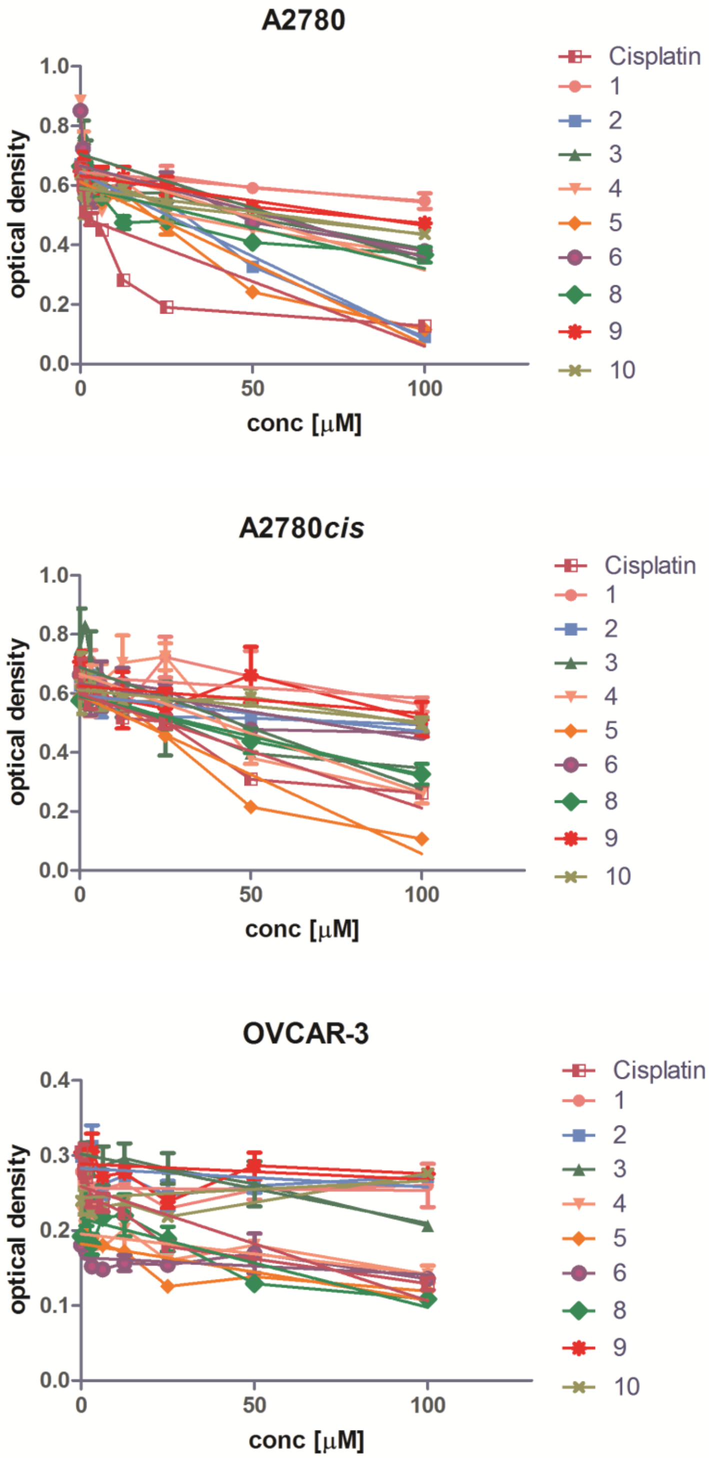


**Figure S14.** Dose-response linear regression of optical density *versus* concentration, in ovarian tumor cells subjected to treatment for 24 hours.

**Table S1.** The cell growth inhibitory capacity of formamidinate ligands (**1**, **2**), dirhodium(II,II) complexes (**3 - 10**) and cisplatin in cancer cell lines (A2780, A2780*cis*, OVCAR-3), analyzed by linear regression hillslope (steepness of the line) significance in the 95% confidence interval. All hillslope values are negative, confirming that a relationship was established between the compounds concentration and the cell growth inhibition; the tendency to inhibit is more evident when $r^2$ fraction is closer to 1, and p value<0.05 indicates statistical significance. Where p>0.05, the growth inhibition is independent of the doses, up to 100μM.

| | | A2780 | | | A2780*cis* | | | OVCAR-3 | | |
|---|---|---|---|---|---|---|---|---|---|---|
| **Compound** | *n* | Hillslope | $r^2$ | p value | Hillslope | $r^2$ | p value | Hillslope | $r^2$ | p value |
| **1** | 0 | -0.0010 ± 0.0002 | 0.50 | 0.0002 | -0.00073 ± 0.00075 | 0.074 | 0.4166* | -0.000032 ± 0.00017 | 0.0025 | 0.8664* |
| **2** | 0 | -0.0056 ± 0.00027 | 0.97 | 0.0138 | -0.0012 ± 0.00044 | 0.39 | 0.0163 | -0.00025 ± 0.00020 | 0.099 | 0.2353* |
| **3** | 2 | -0.0036 ± 0.00065 | 0.65 | <0.0001 | -0.0041 ± 0.00095 | 0.61 | 0.0025 | -0.00092 ± 0.00017 | 0.68 | 0.0009 |
| **4** | 2 | -0.0035 ± 0.00081 | 0.54 | 0.0021 | -0.0040 ± 0.00078 | 0.66 | 0.0013 | -0.00054 ± 0.00023 | 0.28 | 0.0355 |
| **5** | 2 | -0.0055 ± 0.00038 | 0.93 | <0.0001 | -0.0054 ± 0.00042 | 0.93 | <0.0001 | -0.00075 ± 0.00013 | 0.69 | <0.0001 |
| **6** | 2 | -0.0031 ± 0.00070 | 0.56 | <0.0001 | -0.0019 ± 0.00049 | 0.56 | 0.0231 | -0.00023 ± 0.00013 | 0.18 | 0.1012* |
| **8** | 6 | -0.0027 ± 0.00047 | 0.71 | <0.0001 | -0.0027 ± 0.00030 | 0.87 | <0.0001 | -0.0012 ± 0.00024 | 0.65 | 0.0003 |
| **9** | 6 | -0.0017 ± 0.00033 | 0.65 | 0.0037 | -0.00093 ± 0.00071 | 0.12 | 0.1436* | -0.00021 ± 0.00020 | 0.082 | 0.0628* |
| **10** | 6 | -0.0016 ± 0.00036 | 0.57 | 0.0010 | -0.0011 ± 0.00047 | 0.30 | 0.0408 | -0.00023 ± 0.00019 | 0.059 | 0.6159* |
| **Cisplatin** | 1 | -0.0044 ± 0.00099 | 0.58 | <0.0001 | -0.0039 ± 0.00044 | 0.85 | <0.0001 | -0.0015 ± 0.00023 | 0.76 | <0.0001 |

n- number of metal atoms per molecule; $r^2$- correlation coefficient, goodness of fit; * p>0.05, no significant cell growth inhibition.
